# Supplementary material for: Chronic illness and extreme performance: Type 1 diabetes in ultra-endurance
Source: PLoS One. 2025 Dec 19;20(12):e0338637. doi: 10.1371/journal.pone.0338637 (PMC12716752; doi:10.1371/journal.pone.0338637)
Supplement: Attachment — Submitted filename: Response to Reviewers.docx [file pone.0338637.s002.docx]

**Response to Reviewers**

Dear Reviewers,

I would like to sincerely thank both reviewers for the time and effort devoted to reviewing my manuscript, which explores the lived experience of chronic illness—specifically type 1 diabetes—in the context of ultra-endurance sports. Your comments and suggestions have been extremely valuable and have significantly improved the quality of this work.

**Revisions Implemented**

- **Title**: The title has been revised to make it more precise and aligned with PLOS ONE style guidelines.
- **Participant Table**: The table has been enriched with additional data from interviews (age, sex, diabetes duration, technology use, type of race), while respecting ethical constraints. Race names were omitted to preserve anonymity.
- **Ethics and Data Access**: The ethics approval statement now appears only in the Methods section. The institutional contact for data access has been corrected (Data Access Committee, Université de Lorraine).
- **References**: The reference list was checked for retracted articles. Two suggested references could not be retrieved despite extensive searches. However, the concept of *supercrip narratives* was incorporated through the publication by Williams et al. (2021).
- **Sample Justification**: The sample size (n = 13) is consistent with qualitative research standards and reflects the rarity of the target population (likely fewer than one hundred ultra-endurance athletes with type 1 diabetes in France). The gender distribution (15% women) mirrors the reality of ultra-endurance events, where female participation remains limited but is gradually increasing.
- **Reflexivity**: I expanded the description of my dual role as both a physician and a person living with type 1 diabetes, and detailed strategies to minimize bias (reflexive journaling, triangulation with co-authors). This is addressed in the Methods section and acknowledged in the limitations.
- **Clinical Implications**: A paragraph was added to highlight how endocrinologists and primary care physicians can support patients pursuing ultra-endurance goals through individualized guidance and collaborative care.
- **Language and Style**: Several sentences were revised to improve clarity and readability, following your recommendation for a more fluent style.

To facilitate your review, a detailed **point-by-point response table** is provided below.

Once again, thank you for your constructive feedback. I hope this revised version meets your expectations and reflects the quality standards of PLOS ONE.

Best regards

The first author on behalf of the team

Point-by-Point Response Table :

| **Reviewer Comment** | **Response** |
| --- | --- |
| **Title: Please revise the manuscript title to remove redundant wording.** | The title was revised to *“Chronic Illness and Extreme Performance: Type 1 Diabetes in Ultra-Endurance”* to improve clarity and comply with PLOS ONE style. |
| **Participant Table: Add pseudonymized data and relevant characteristics.** | The table was expanded to include age, sex, diabetes duration, technology use, and type of race, while respecting anonymity and ethical constraints. |
| **Reflexivity: Elaborate on the dual role of the researcher and strategies to limit bias.** | A dedicated paragraph was added in the Methods section detailing the principal investigator’s dual role and mitigation strategies (reflexive journaling, triangulation). |
| **Sample Composition: Discuss size and gender bias.** | Added justification in Strengths and Limitations: sample size aligns with qualitative standards and reflects the rarity of the population; gender distribution mirrors ultra-endurance participation trends. |
| **Clinical Implications: How can endocrinologists support these** | A paragraph was added in Practical Implications, highlighting the role of endocrinologists and general practitioners in supporting safe participation through individualized guidance and collaborative care. |
| **References: Include suggested citations.** | Integrated Williams et al. (2021) for *supercrip narratives*. Other suggested references could not be retrieved despite extensive searches. |
| **Language and Style: Improve** | Several sentences were revised for clarity and readability throughout the manuscript. |
